# Ankle Function and Donor-Site Morbidity Following Peroneus Longus Graft Harvesting with or Without Tenodesis to Peroneus Brevis in Anterior Cruciate Ligament Reconstruction

**DOI:** 10.3390/jcm15072577

**Published:** 2026-03-27

**Authors:** Firat Dogruoz, Mustafa Kursat Sari, Mehmet Baris Ertan, Ali Ergun, Serkan Gurcan, Ozkan Kose

**Affiliations:** 1 Department of Orthopedics and Traumatology, Antalya Education and Research Hospital, University of Health Sciences, Antalya 07100, Turkey; 2 Department of Orthopedics and Traumatology, Sehit Kamil State Hospital, Gaziantep 27500, Turkey; 3 Orthopedics and Traumatology Clinic, Medikum Private Hospital, Antalya 07350, Turkey; 4 Private Practice, Gurcan Orthopedics Clinic, Cevizlik, Istanbul 34365, Turkey

**Keywords:** anterior cruciate ligament reconstruction, peroneus longus tendon, tenodesis, donor site morbidity, ankle joint, radiography, flatfoot

## Abstract

**Background/Objectives:** The peroneus longus tendon (PLT) is increasingly used as an autograft for anterior cruciate ligament reconstruction (ACLR). However, during PLT harvest, the necessity of distal peroneus longus-to-peroneus brevis (PL-to-PB) tenodesis for the potential preservation of donor ankle function and medial longitudinal arch alignment remains unclear. This study compared ankle function, donor-site morbidity, complications, and weight-bearing radiographic foot alignment after PLT harvest with and without distal tenodesis. **Methods:** Between January 2020 and December 2024, 92 primary ACLR cases using an ipsilateral PLT autograft were retrospectively screened; 60 patients with available bilateral weight-bearing comparative foot radiographs were included and categorized into a tenodesis group (*n* = 30) or a non-tenodesis group (*n* = 30). Ankle outcomes included American Orthopedic Foot and Ankle Society (AOFAS) Ankle–Hindfoot and Foot and Ankle Disability Index (FADI) scores, ankle range of motion (ROM), and donor-site complications. Radiographic alignment was assessed using Meary’s angle and calcaneal pitch angle on bilateral weight-bearing lateral foot radiographs, including side-to-side differences. **Results:** Follow-up duration was comparable between groups (18.5 ± 4.4 vs. 16.8 ± 3.4 months, *p* = 0.113). No patient demonstrated clinically relevant loss of ankle range of motion or strength at final follow-up. AOFAS (97.3 ± 4.9 vs. 95.0 ± 5.5, *p* = 0.078) and FADI (96.8 ± 5.2 vs. 95.3 ± 5.5, *p* = 0.091) scores were similarly high in the tenodesis and non-tenodesis groups, respectively. Sural nerve sensory disturbance occurred in 6/30 (20.0%) versus 5/30 (16.7%) patients (*p* = 0.739), and no harvest-site infection was observed. On weight-bearing radiographs, Meary’s angle and calcaneal pitch angle did not differ significantly between groups on the operated side (Meary: 7.99 ± 6.76 vs. 4.76 ± 6.32°, *p* = 0.061; calcaneal pitch: 23.19 ± 5.94 vs. 21.41 ± 4.64°, *p* = 0.201) or intact side (Meary: 7.05 ± 6.89 vs. 5.36 ± 6.11°, *p* = 0.320; calcaneal pitch: 23.33 ± 5.43 vs. 22.00 ± 4.48°, *p* = 0.305). Side-to-side differences were small and comparable (Δ Meary: 0.94 ± 3.97 vs. −0.60 ± 3.58°, *p* = 0.120; Δ calcaneal pitch: −0.14 ± 3.35 vs. −0.59 ± 3.29°, *p* = 0.603). **Conclusions:** Distal PL-to-PB tenodesis did not appear to provide measurable advantages in donor-ankle patient-reported outcomes or weight-bearing radiographic foot alignment compared with no tenodesis after PLT harvest for ACLR.

## 1. Introduction

Anterior cruciate ligament (ACL) injuries are among the most common ligamentous injuries of the knee, particularly in physically active individuals [1]. ACL reconstruction is often necessary to restore joint stability and function, and the choice of graft is critical to surgical success. While hamstring tendons and bone–patellar tendon–bone autografts have traditionally been preferred, alternative graft options have gained popularity in recent years [2,3]. Among these, the peroneus longus tendon (PLT) has emerged as a reliable and biomechanically strong autograft for ACL reconstruction [4,5]. The use of PLT as an autograft offers several potential advantages, particularly in minimizing donor site morbidity. Unlike hamstring harvesting, which may compromise knee flexor strength, or patellar tendon harvesting, which may lead to anterior knee pain, PLT harvesting is associated with the preservation of knee-related muscle function and potentially a lower incidence of donor-site complications [2,6,7]. Moreover, the anatomical length, tensile strength, and ease of harvest make PLT a compelling graft option in both primary and revision ACL surgeries [4,5,8].

Nevertheless, PLT harvesting presents unique considerations for managing the distal tendon stump. PLT was first introduced for ACL reconstruction in 2008 by Kerimoglu et al., who reported performing the procedure without distal tenodesis [9]. In subsequent years, many surgeons adopted PL-to-PB tenodesis after graft harvest to preserve eversion strength, lateral ankle stability, and first-ray function [10,11]. This rationale is based on the recognized biomechanical role of the peroneus longus in first-ray plantarflexion and dynamic support of the medial longitudinal arch through its course beneath the foot and insertion at the base of the first metatarsal and medial cuneiform. However, the necessity of this additional step remains debated [12,13]. While tenodesis is theoretically appealing, others argue that the peroneus brevis and surrounding musculature may provide sufficient compensation even without formal fixation and that routine tenodesis may not translate into measurable clinical benefit [14,15,16,17]. Importantly, despite the growing use of PLT autografts, direct comparative clinical data evaluating tenodesis versus no tenodesis remain limited, and the potential effect of distal tenodesis on donor-ankle function and radiographic foot alignment is not well established. To our knowledge, only two clinical studies have specifically examined the role of distal PL-to-PB tenodesis after PLT harvest [12,13]. Therefore, the present study aimed to compare ankle function, donor-site morbidity, and weight-bearing radiographic parameters of the medial longitudinal arch in patients undergoing PLT harvest with or without distal PL-to-PB tenodesis during ACLR. We hypothesized that the tenodesis and non-tenodesis groups would demonstrate comparable donor-ankle functional outcomes, including American Orthopedic Foot and Ankle Society (AOFAS) Ankle–Hindfoot and Foot and Ankle Disability Index (FADI) scores, ankle range of motion (ROM), and donor-site complications, as well as comparable radiographic alignment parameters, specifically Meary’s angle, calcaneal pitch angle, and side-to-side differences on bilateral weight-bearing lateral foot radiographs.

## 2. Materials and Methods

### 2.1. Patients, Study Design, and Sample Size Calculation

This retrospective comparative cohort study was conducted at the University of Health Sciences Antalya Education and Research Hospital. The institutional surgical database was reviewed to identify all consecutive patients who underwent primary ACLR using an ipsilateral PLT autograft between January 2020 and December 2024. During this period, 92 patients who received a PLT autograft for ACLR were identified. Because the primary objective of the present study was to assess donor-site morbidity following PLT harvesting, including foot function and foot alignment, only patients with bilateral weight-bearing comparative foot radiographs available at follow-up were eligible for inclusion. Based on this predefined criterion, 60 patients constituted the final study cohort. Patients were subsequently categorized according to whether a PL-to-PB tenodesis had been performed at the time of graft harvest. Accordingly, the cohort was divided into a tenodesis group (*n* = 30) and a non-tenodesis group (*n* = 30).

An a priori sample size calculation was performed using the AOFAS Ankle–Hindfoot score as the primary outcome. A between-group difference of 5 points was considered clinically meaningful, in line with published estimates of the minimal clinically important difference (MCID) for the AOFAS Ankle–Hindfoot Scale (approximately 4.1–7.8 points) [18]. Assuming a two-sided α of 0.05, 80% power, and an anticipated standard deviation of 5.2 points, the minimum required sample size was 17 patients per group. Accordingly, the available sample of 30 patients per group (total *n* = 60) provided sufficient statistical power for the planned between-group comparisons.

Patients were included if they underwent primary ACLR with an ipsilateral PLT autograft, had at least 12 months of follow-up, and completed the standardized postoperative rehabilitation and follow-up protocol. Exclusion criteria were previous surgery, fracture, or significant trauma to the ipsilateral ankle/foot; pre-existing ankle/foot deformity, inflammatory arthropathy, neuromuscular disease, or symptomatic peroneal tendon disorder; concomitant conditions or procedures likely to substantially affect gait or ankle loading (e.g., major contralateral lower-limb pathology or complex multiligament reconstructions); and incomplete medical records or missing outcome data.

Data were extracted from electronic medical records using a standardized data collection form. Baseline demographic and clinical variables included age, sex, operated side, anthropometric data [weight, height, and body mass index (BMI)], mechanism of injury, tobacco use, and American Society of Anesthesiologists (ASA) classification. Perioperative variables included operative time, length of hospital stay, concomitant meniscal status and procedures (medial and lateral meniscus classified as intact, repair, or partial meniscectomy), the PLT harvesting approach (single inframalleolar, single supramalleolar, or double-incision technique), graft preparation method (double- vs. triple-stranded), and intraoperatively measured graft diameter. The study protocol was approved by the University of Health Sciences, Antalya Education and Research Hospital Ethics Committee (approval date: 3 July 2025; No.: 11/2) and was conducted in accordance with the Declaration of Helsinki. Informed consent was obtained from all participants.

### 2.2. ACL Reconstruction Technique

All procedures were performed with the patient in the supine position under general or spinal anesthesia with tourniquet control. After standard portal placement, a routine diagnostic arthroscopy was carried out to confirm anterior cruciate ligament rupture and to evaluate associated intra-articular pathology. The femoral tunnel was created using an anteromedial portal technique in all cases, and femoral fixation was achieved with a suspensory cortical button device [1]. Tibial fixation was performed using a bioabsorbable interference screw in all patients, and additional tibial fixation was reinforced with a U-staple when deemed necessary. Concomitant meniscal and chondral lesions were addressed after graft passage through the femoral and tibial tunnels and prior to final tibial fixation. At the conclusion of the procedure, a suction drain was inserted, and a compressive dressing was applied. The procedures were performed by four orthopedic surgeons within the same institutional sports surgery team. Although the operations were not limited to a single surgeon, surgical decision-making, operative workflow, graft preparation, fixation principles, and postoperative rehabilitation were based on the department’s shared clinical practice pattern.

### 2.3. Graft Harvesting Technique

PLT harvesting was performed using one of three incision strategies: a single inframalleolar incision, a single supramalleolar incision, or a double-incision approach combining inframalleolar and supramalleolar incisions [4,6,8]. Importantly, PL-to-PB tenodesis was performed or omitted as a separate step, independent of the incision strategy. After standard sterile preparation and draping, anatomical landmarks, including the lateral malleolus and the course of the peroneal tendons toward the base of the fifth metatarsal, were marked on the skin. In all techniques, the peroneal tendon sheath was opened longitudinally, and the PLT was identified and differentiated from the PB tendon in the peroneal compartment. Care was taken to protect the surrounding soft tissues, including the superficial sensory branches of the sural nerve. A traction suture was placed on the PLT to facilitate controlled handling. The PLT was then released distally at the planned level, and proximal harvesting was performed using a tendon stripper advanced proximally along the tendon’s longitudinal axis with the ankle maintained in a neutral position.

**Figure 1:**
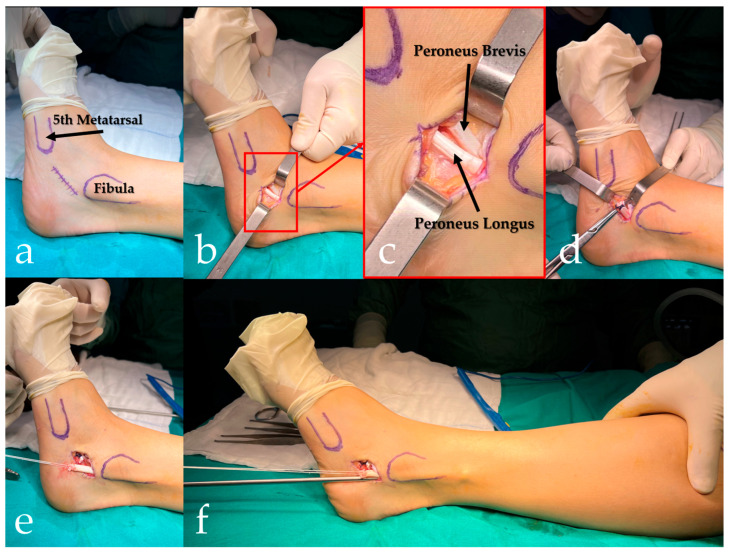
Single inframalleolar incision technique with peroneus longus–to–peroneus brevis tenodesis. (**a**) Surface landmarking on the lateral aspect of the ankle and hindfoot (distal fibula and base of the fifth metatarsal). (**b**) Inframalleolar longitudinal incision with exposure of the peroneal tendons within the sheath. (**c**) Magnified view of the area (red rectangle) outlined in (**b**), demonstrating the peroneus brevis (PB) and peroneus longus tendon (PLT). (**d**) Side-to-side PLT-to-PB tenodesis was performed through the inframalleolar incision prior to tendon transection. (**e**) Introduction of the tendon stripper for proximal graft harvesting through the same inframalleolar incision. (**f**) Retrieval of the harvested PLT graft after proximal stripping.

In the single inframalleolar technique, a short longitudinal incision was made distal to the lateral malleolus over the peroneal tendon course [8]. Through this incision, the PLT and PBT were exposed within the sheath, the PLT was isolated, and the distal division was performed. The tendon stripper was then introduced through the same incision and advanced proximally to harvest the graft, which was delivered through the inframalleolar wound (Figure 1).

In the single supramalleolar technique, a longitudinal incision was made 2–3 cm proximal to the tip of the lateral malleolus along the posterolateral aspect of the distal fibula, corresponding to the peroneal tendon path in the retromalleolar region. The tendon sheath was opened, and the PLT was isolated at this level. After distal release and traction suture placement, the tendon stripper was advanced proximally, and the harvested graft was retrieved through the supramalleolar incision.

**Figure 2:**
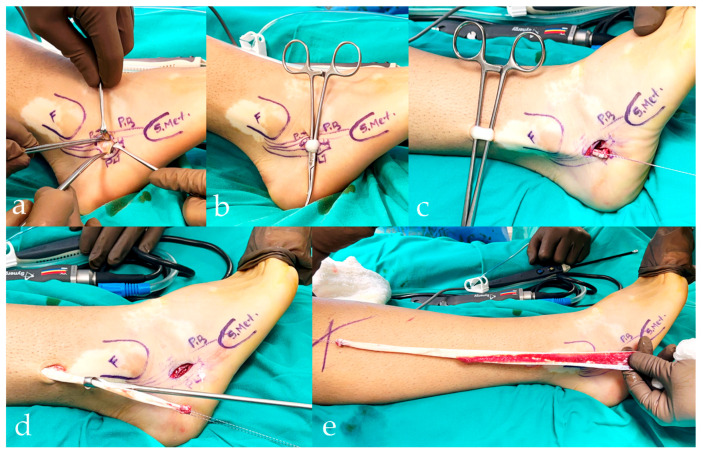
Double-incision technique without peroneus longus–to–peroneus brevis tenodesis. (**a**) Inframalleolar incision with exposure of the peroneal tendons and identification of the peroneus longus tendon (PLT) and peroneus brevis (PB). (**b**) Distal control of the PLT through the inframalleolar window prior to transection. (**c**) After distal division, the PLT is prepared for proximal stripping; no PL-to-PB tenodesis is performed. (**d**) A second supramalleolar incision is created for proximal retrieval when passage of the tendon stripper through the retromalleolar tunnel is limited. (**e**) The harvested PLT graft is delivered through the supramalleolar incision following proximal stripping.

In the double-incision technique, an inframalleolar incision was first used to expose the peroneal tendons, confirm tendon identity, and divide the PLT distally under direct visualization. A second supramalleolar incision was then made to facilitate graft retrieval. The proximally harvested tendon was delivered through the supramalleolar incision, and graft harvesting was completed from this proximal wound. This approach was primarily used when advancement of the tendon stripper from the distal incision was impeded at the retromalleolar level; in such cases, the additional supramalleolar incision allowed proximal retrieval of the graft while minimizing manipulation within the retromalleolar tunnel and reducing the risk of iatrogenic injury to the peroneal retinaculum (Figure 2).

Subsequent to graft harvest, the peroneal tendon sheath was irrigated and closed as deemed appropriate, while the skin was closed in a layered fashion. In cases where PL-to-PB tenodesis was performed, a side-to-side tenodesis using nonabsorbable sutures was completed before the transection and separation of the PLT. This ensured the continuity of the distal peroneal unit prior to graft harvest. In instances where tenodesis was not performed, the PLT was divided according to the planned harvesting technique, and the distal stump was left without tendon-to-tendon fixation, while all other steps were standardized.

No formal randomization was used for distal PL-to-PB tenodesis. Instead, patients were categorized retrospectively based on whether tenodesis had been performed, and the decision to perform or omit tenodesis reflected the operating surgeon’s preferred graft-harvesting technique.

### 2.4. Postoperative Rehabilitation

All patients followed the same institution-based standardized postoperative rehabilitation protocol, independent of whether distal PL-to-PB tenodesis had been performed. Rehabilitation was supervised by the same physiotherapy team and advanced according to uniform clinical criteria. Early rehabilitation focused on controlling pain and edema, restoring full knee extension, and progressively recovering knee flexion, followed by strengthening, proprioceptive training, running, and sport-specific exercises. In patients with concomitant meniscal repair, however, knee rehabilitation was modified to protect the repair site; knee flexion was initially restricted to 30° and then gradually increased, and weight-bearing was postponed until 4–6 weeks postoperatively. No ankle immobilization or restriction was used in either group. Ankle ROM was unrestricted, and weight-bearing was allowed as tolerated per the postoperative protocol.

### 2.5. Functional Assessments

All patients in both groups were evaluated in person using a standardized follow-up protocol. To minimize inter-rater variability, all interviews, clinical examination maneuvers, range-of-motion measurements, and strength assessments were performed by the same orthopedic surgeon with five years of experience. Knee-related outcomes were assessed using the Lysholm Knee Score (LKS) obtained preoperatively and at final follow-up [19]. Bilateral thigh circumference (measured at the level of the superior pole of the patella) and maximal calf circumference were recorded to document side-to-side differences. Knee stability was evaluated using the Lachman test, with laxity graded from Grade 0 to Grade 3 (Grade 0 indicating a negative test). Knee ROM was measured bilaterally with a goniometer in the supine position using standard anatomical landmarks, and clinically relevant flexion or extension loss was defined a priori as a contralateral-referenced deficit of ≥5°. Functional performance was evaluated using the single-leg hop test on both limbs. After standardized familiarization, three trials were performed for each limb, and the best distance was recorded; the limb symmetry index (LSI) was calculated as (involved/uninvolved) × 100 [20].

Donor-site function and ankle-related outcomes after peroneus longus tendon harvest were assessed using the AOFAS Ankle–Hindfoot Score and the FADI [21,22]. Ankle range of motion was examined bilaterally, including plantarflexion, dorsiflexion, inversion, and eversion, and any side-to-side motion loss was documented. Ankle strength in plantarflexion, dorsiflexion, inversion, and eversion was assessed clinically by manual muscle testing during the standardized physical examination; no instrumented dynamometric assessment was performed. A focused neurovascular examination was performed at each follow-up visit, and donor-site complications were systematically documented, including sensory deficits consistent with sural nerve injury and wound-related complications at the harvest site.

### 2.6. Radiographic Assessment and Measurements

**Figure 3:**
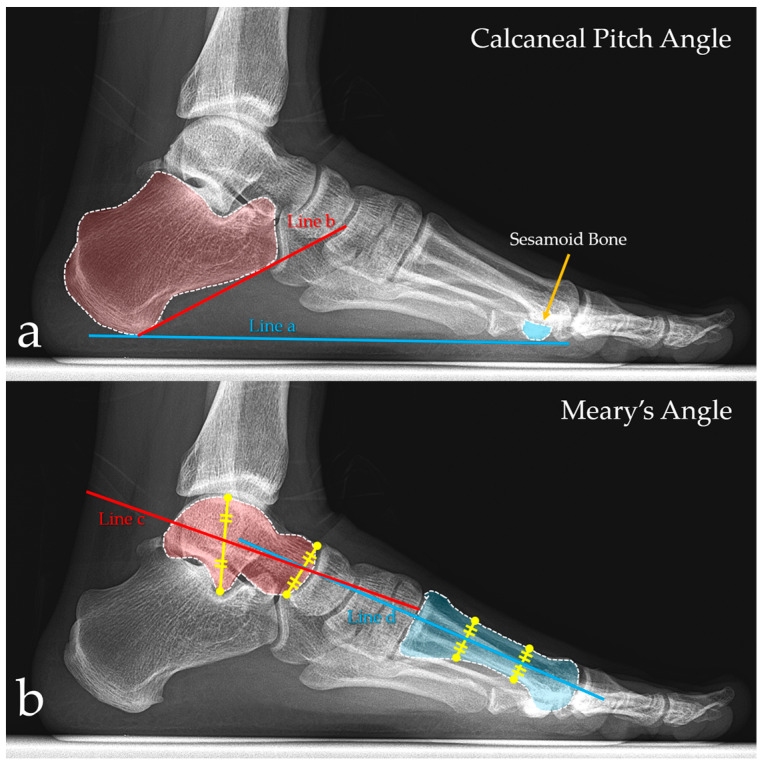
Radiographic assessment of the medial longitudinal arch on weight-bearing lateral foot radiographs. (**a**) Calcaneal pitch angle measurement: the angle between the floor line (Line a) and the line along the inferior surface of the calcaneus (Line b). The sesamoid bone is shown for orientation. (**b**) Meary’s angle (talo–first metatarsal angle) measurement: the angle between the longitudinal axis of the talus (Line c) and the longitudinal axis of the first metatarsal (Line d). Yellow lines are used to locate the longitudinal axis of the talus and the first metatarsal bone.

Radiographic evaluation of the medial longitudinal arch was performed using bilateral weight-bearing lateral foot radiographs. All radiographs were obtained with patients standing in a comfortable, neutral stance, with full body weight evenly distributed on both feet. Images were acquired in a standardized manner in accordance with the institutional protocol and stored in the institutional Picture Archiving and Communication Systems (PACS). Two established radiographic parameters were used to assess foot arch alignment: the calcaneal pitch angle and Meary’s angle (talo–first metatarsal angle) [23,24,25]. These two parameters were selected because the primary radiographic aim of the study was to assess preservation of the medial longitudinal arch on standardized weight-bearing lateral radiographs rather than to perform a comprehensive multiplanar evaluation of overall foot alignment. All measurements were performed on the weight-bearing lateral view. The calcaneal pitch angle was defined as the angle between a reference line representing the weight-bearing surface (floor line; Line a) and a line drawn along the inferior surface of the calcaneus (Line b), extending from the most plantar point of the calcaneal tuberosity toward the plantar aspect of the anterior calcaneus. The angle formed by the intersection of these two lines was recorded as the calcaneal pitch angle (Figure 3a). Meary’s angle was defined as the angle between the longitudinal axis of the talus (Line c) and the longitudinal axis of the first metatarsal (Line d). The talar axis was determined by bisecting the talar body and neck, whereas the first metatarsal axis was determined by connecting the mid-diaphyseal points of the first metatarsal. The angle between these two axes was recorded as Meary’s angle (Figure 3b).

All measurements were performed independently by two observers (M.B.E., M.K.S.) who were blinded to the clinical outcomes and group allocation. Measurements were obtained digitally using a dedicated imaging workstation/software (Sectra IDS 7; Sectra AB, Linköping, Sweden). To assess intraobserver reliability, all measurements were repeated after a two-week interval. Inter- and intraobserver reliability were quantified using the intraclass correlation coefficient (ICC) based on a two-way random-effects model with absolute agreement for single measurements (ICC [2,1]). ICC values were interpreted as <0.50 (poor), 0.50–0.75 (moderate), 0.75–0.90 (good), and >0.90 (excellent) reliability [26]. Reliability analyses are presented in Table 1. As inter- and intraobserver agreement was good to excellent, the mean of the four measurements obtained for each radiographic variable (two observers × two sessions) was used for subsequent analyses.

### 2.7. Statistical Analysis

Statistical analyses were performed using IBM SPSS Statistics Base for Windows, V.23.0 (IBM Corp., Armonk, NY, USA). Continuous variables were evaluated for normality using the Shapiro–Wilk test and visual inspection of histograms and Q–Q plots. Normally distributed data are presented as mean ± standard deviation, whereas non-normally distributed data are presented as median (interquartile range). Categorical variables are reported as counts and percentages. Between-group comparisons (tenodesis vs. non-tenodesis) were conducted using the independent-samples *t*-test for normally distributed continuous variables and the Mann–Whitney U test for non-normally distributed continuous variables. Categorical variables were compared using the chi-square test or Fisher’s exact test, as appropriate. Within-group preoperative–postoperative comparisons were performed using the paired-samples *t*-test or the Wilcoxon signed-rank test, depending on data distribution. For radiographic outcomes, operated-side and contralateral-side measurements were compared within groups using paired-samples tests, and side-to-side differences (Δ = operated − intact) were calculated and compared between groups. Intraclass correlation coefficients (ICC [2,1]) with 95% confidence intervals were used to assess inter- and intraobserver reliability of radiographic measurements. For the main continuous between-group comparisons, effect sizes were calculated using Hedges’ g to quantify the magnitude of differences independently of statistical significance. No formal adjustment for multiple comparisons was applied; therefore, analyses other than the prespecified primary outcome should be interpreted as secondary or exploratory. All tests were two-tailed, and statistical significance was set at *p* < 0.05.

## 3. Results

A total of 92 patients who underwent primary ACL reconstruction with a peroneus longus tendon autograft between January 2020 and December 2024 were screened. Sixty patients with bilateral weight-bearing comparative foot radiographs available at follow-up met the inclusion criteria and constituted the final cohort. Patients were categorized into the tenodesis (*n* = 30) and non-tenodesis (*n* = 30) groups. Baseline demographic and clinical characteristics were comparable between the groups, with no statistically significant differences in age, sex distribution, operated side, anthropometric variables (weight, height, BMI), mechanism of injury, tobacco use, or ASA classification (Table 2).

Perioperative variables, including operative time and length of hospital stay, did not differ between the groups. Concomitant meniscal status/procedures (medial and lateral) and the distribution of PLT harvesting approaches (single inframalleolar, single supramalleolar, double-incision) were also comparable. In contrast, graft preparation technique and graft diameter differed between groups; the tenodesis group more frequently underwent double-stranded graft preparation and had a smaller mean graft diameter than the non-tenodesis group (Table 3).

Both groups demonstrated significant within-group improvement in Lysholm Knee Score from the preoperative assessment to final follow-up. The magnitude of improvement (Δ LKS) was greater in the tenodesis group. However, objective stability (Lachman grading), range-of-motion deficits (flexion/extension loss), thigh and calf circumference differences, single-leg hop limb symmetry index, and re-rupture rates were comparable between groups (Table 4).

No patient in either group demonstrated clinically relevant loss of ankle range of motion or strength in any plane at final follow-up. Patient-reported ankle/foot outcomes (AOFAS and FADI) were high in both groups, with no statistically significant between-group differences. Donor-site complications were uncommon; no harvest-site infections were recorded, and sural nerve sensory disturbance occurred at similar rates in both groups (Table 5).

On weight-bearing radiographs, Meary’s angle and calcaneal pitch angle did not differ significantly between the tenodesis and non-tenodesis groups on either the operated or intact side. Within each group, operated-side angles were not significantly different from the contralateral intact side (Table 6). The side-to-side differences (Δ angles: operated minus intact) were small and did not differ between groups (Table 7). Effect size estimates for the main ankle and radiographic continuous outcomes were small overall, indicating that the between-group differences were limited in magnitude.

## 4. Discussion

In this retrospective comparative cohort of patients undergoing ACL reconstruction with a PLT autograft, the principal finding was that performing a distal PL-to-PB tenodesis did not result in superior ankle function or radiographic foot alignment at short- and mid-term follow-up. Patient-reported ankle outcomes were high in both groups, and neither clinically meaningful loss of ankle motion nor strength deficit was detected on examination. Likewise, weight-bearing radiographs demonstrated no group-related differences in Meary’s angle or calcaneal pitch angle on the operated or contralateral side, and side-to-side (operated–intact) differences were small and comparable. Taken together, these results suggest that routine tenodesis after PLT harvest may not provide additional clinical or radiographic benefit in many patients, as preservation of ankle function and medial longitudinal arch alignment appeared similar with or without tenodesis.

From a biomechanical perspective, concern about donor-site morbidity after PLT harvest primarily stems from the tendon’s contribution to first-ray plantarflexion and midfoot stabilization, functions traditionally linked to maintaining the medial longitudinal arch during stance and push-off [14,15]. However, the peroneal complex functions as a synergistic unit, and several lines of evidence suggest that removal of the PLT does not necessarily lead to clinically relevant arch collapse. First, experimental work has shown that, for generating the eversion moment that counterbalances inversion forces, the PB mechanism may be more effective than the PL mechanism, implying that preservation of the PB could mitigate potential hindfoot valgus/instability even when PL is harvested [27]. Second, clinical studies assessing donor ankle function after PLT harvest—using functional scores and objective measures such as eversion/first-ray plantarflexion strength and gait parameters—have generally reported minimal functional compromise, suggesting that compensatory activation of the PB and other plantar flexors/evertors can maintain overall foot mechanics [11,28]. In addition, broader syntheses of the available evidence indicate that although small statistical changes in ankle outcome scores may be detectable, the magnitude is typically too small to be clinically meaningful, supporting the concept that the foot–ankle complex adapts well following PL harvest [29]. Finally, studies incorporating objective assessments of foot morphology and gait after partial or full PL harvest have similarly suggested no major structural deterioration of the arch, although subtle changes in specific gait cycle parameters may occur [12,30].

This biomechanical rationale is consistent with our radiographic findings. On bilateral weight-bearing lateral foot radiographs, neither Meary’s angle nor the calcaneal pitch angle differed between the tenodesis and non-tenodesis groups on either the operated or contralateral side. More importantly, the within-patient side-to-side differences were small for both parameters and did not differ between groups. Although Δ Meary’s angle showed a slight positive tendency in the tenodesis group and a slight negative tendency in the non-tenodesis group, this difference was not statistically significant, and Δ calcaneal pitch values were similarly minimal. In addition, one-sample testing demonstrated that the Δ values were not significantly different from zero within either group, further supporting the absence of a systematic radiographic shift toward arch collapse after PLT harvest. Taken together, these findings suggest that the medial longitudinal arch can be preserved on weight-bearing imaging following PLT harvest, likely through compensatory contributions of the PB and other dynamic stabilizers, and that this apparent adaptation may occur independently of distal PL-to-PB tenodesis. From a clinical perspective, these findings suggest that PLT harvest did not produce a radiographically detectable tendency toward progressive arch collapse or symptomatic pes planus during the follow-up period and that distal PL-to-PB tenodesis did not appear to provide additional structural protection in this regard.

Clinical evidence to date generally supports minimal donor-site morbidity after PLT harvest, but most series have historically included routine PL-to-PB tenodesis, making it difficult to isolate the true incremental value of tenodesis. Systematic reviews and meta-analyses consistently show that postoperative ankle scores (most commonly AOFAS and FADI) remain high, with only small, statistically detectable changes that are typically unlikely to be clinically meaningful [29,31]. Among individual clinical studies, objective testing has often failed to demonstrate meaningful side-to-side deficits in key functions attributed to the peroneus longus, such as eversion and first-ray plantarflexion strength, while patient-reported ankle outcomes remain excellent [11,32]. Importantly, more recent data specifically addressing tenodesis versus no tenodesis suggest that tenodesis may not confer measurable benefit in validated ankle outcomes: in a large prospective cohort, Arora et al. reported no meaningful differences in AOFAS or FADI across follow-up intervals, with only a very small early advantage for the no-tenodesis group that was not clinically relevant [12]. Likewise, series that explicitly performed without peroneal tenodesis have reported excellent donor-ankle function on the AOFAS with low rates of sensory symptoms, supporting the notion that acceptable ankle outcomes can be achieved even when the PL stump is not sutured to PB [13]. In the present series, these injuries were sensory in nature and did not lead to major functional impairment or require additional treatment, but they remain clinically relevant because persistent numbness or dysesthesia may affect patient comfort and satisfaction. At the same time, a minority of studies using more sensitive objective tools (e.g., dynamometry, pedobarography, or gait/postural-control analyses) have identified subtle alterations, such as evertor/first-ray plantarflexion weakness or pressure distribution changes, despite reassuring PROMs, suggesting that traditional scoring systems may not fully capture small biomechanical adaptations [28,33]. In this context, our findings, showing similarly high AOFAS/FADI scores and no difference in donor ankle complications or radiographic arch parameters between groups, suggest that distal tenodesis does not appear to translate into superior ankle outcomes in routine clinical follow-up. Moreover, the small effect sizes observed for the main ankle and radiographic outcomes suggest that the between-group differences were limited not only statistically but also in practical terms, consistent with prior studies reporting minimal donor-site disadvantage after PLT harvest. Although the present findings are reassuring from a general clinical perspective, their applicability to high-demand populations such as competitive athletes should be interpreted with caution, because subtle donor-site deficits may become more relevant under conditions of greater functional demand and were not assessed using performance-based biomechanical testing in this study. Moreover, the uniformly high postoperative AOFAS and FADI scores in both groups also raise the possibility of a ceiling effect, which may have limited the ability of these PROMs to detect subtle between-group differences.

Despite the clinically relevant question addressed, several strengths and limitations of the present study should be considered. A key strength is that donor-site effects were evaluated using bilateral weight-bearing radiographs in addition to clinical assessment, allowing an objective appraisal of medial longitudinal arch alignment after peroneus longus harvest in a cohort directly comparing tenodesis versus no tenodesis. Furthermore, radiographic measurements were obtained using a standardized technique and demonstrated good-to-excellent reliability, supporting the robustness of the imaging-based outcomes.

On the other hand, the retrospective design inherently limits causal inference, and the findings should therefore be interpreted as comparative observational data rather than evidence of causality. Restricting inclusion to patients with available follow-up weight-bearing radiographs may have introduced selection bias. A follow-up duration of approximately 16–18 months may also be insufficient to capture longer-term donor-site functional adaptations or delayed radiographic changes in foot alignment. In addition, group allocation was not randomized and was based on surgeon preference, which may have introduced treatment-selection bias and residual confounding. The observed intergroup differences in graft diameter and graft preparation technique should likewise be considered potential procedural confounders when interpreting the comparative findings. Furthermore, no formal correction for multiple comparisons was applied; therefore, secondary and exploratory analyses should be interpreted with caution because of the potential for type I error inflation. In addition, the significant between-group difference in preoperative Lysholm scores may have influenced the interpretation of postoperative improvement in knee-related outcomes. Although validated patient-reported outcome measures were used, more sensitive objective assessments (e.g., isokinetic dynamometry, pedobarography, or instrumented gait analysis) were not performed and could have detected subtle biomechanical adaptations not captured by PROMs. Another limitation is that the procedures were performed by multiple surgeons rather than a single operator. Although all surgeons worked within the same institutional team and followed similar surgical and rehabilitation principles, some degree of inter-surgeon variability cannot be completely excluded. Finally, radiographic assessment was limited to Meary’s angle and calcaneal pitch on lateral views. Although these are widely accepted parameters, complementary measurements or advanced imaging could further refine the evaluation of foot morphology. Moreover, although radiographic measurements were obtained using a standardized technique and demonstrated good-to-excellent interobserver reliability, potential measurement bias related to patient positioning, radiographic projection, and landmark identification cannot be completely ruled out.

## 5. Conclusions

In patients undergoing primary ACL reconstruction with a PLT autograft, distal PL-to-PB tenodesis did not appear to provide superior donor-ankle outcomes compared with no tenodesis. Patient-reported ankle function remained high in both groups; no major donor-site deficits in ankle ROM or strength were observed, and donor-site complications were limited. Importantly, weight-bearing radiographic parameters of medial longitudinal arch alignment were preserved and did not differ between groups or between operated and contralateral sides. These findings suggest that routine distal tenodesis after PLT harvest may not provide an additional clinical or radiographic advantage for maintaining ankle function and radiographic foot alignment in the short- and mid-term in this cohort, although prospective studies incorporating objective biomechanical assessments are warranted to confirm these findings in high-demand athletic populations.

## Figures and Tables

**Table 1:** Intra- and interobserver reliability for radiographic measurements.

Measurement	Comparison	ICC [2,1]	95% CI	Interpretation
Meary’s angle (Intact)	Intra-observer reliability			
	Obs. A t_1_ vs. t_2_	0.923	0.889–0.943	Excellent
	Obs. B t_1_ vs. t_2_	0.961	0.936–0.976	Excellent
	Inter-observer reliability			
	Obs. A t_1_ vs. Obs. B t_1_	0.834	0.760–0.882	Good
	Obs. A t_2_ vs. Obs. B t_2_	0.970	0.955–0.980	Excellent
Meary’s angle (Operated)	Intra-observer reliability			
	Obs. A t_1_ vs. t_2_	0.964	0.943–0.977	Excellent
	Obs. B t_1_ vs. t_2_	0.960	0.934–0.976	Excellent
	Inter-observer reliability			
	Obs. A t_1_ vs. Obs. B t_1_	0.928	0.885–0.954	Excellent
	Obs. A t_2_ vs. Obs. B t_2_	0.961	0.936–0.977	Excellent
Calcaneal pitch angle (Intact)	Intra-observer reliability			
	Obs. A t_1_ vs. t_2_	0.987	0.978–0.992	Excellent
	Obs. B t_1_ vs. t_2_	0.981	0.969–0.989	Excellent
	Inter-observer reliability			
	Obs. A t_1_ vs. Obs. B t_1_	0.942	0.906–0.964	Excellent
	Obs. A t_2_ vs. Obs. B t_2_	0.987	0.978–0.992	Excellent
Calcaneal pitch angle (Operated)	Intra-observer reliability			
	Obs. A t_1_ vs. t_2_	0.988	0.980–0.993	Excellent
	Obs. B t_1_ vs. t_2_	0.978	0.964–0.986	Excellent
	Inter-observer reliability			
	Obs. A t_1_ vs. Obs. B t_1_	0.968	0.948–0.981	Excellent
	Obs. A t_2_ vs. Obs. B t_2_	0.988	0.980–0.993	Excellent

Abbreviations, ICC: Intraclass Correlation Coefficient, CI: Confidence Interval, Obs.: Observer, t_1_: First time, t_2_: Second time.

**Table 2:** Demographic and clinical characteristics of the patients.

Variables	Tenodesis Group (*n* = 30)	Non-Tenodesis Group (*n* = 30)	*p*-Value
Age (years ± SD)	29.3 ± 10.6	27.3 ± 9.2	0.559 ^1^
Sex (Male vs. Female, *n*, %)	22 (73.3%) vs. 8 (26.7%)	23 (76.7%) vs. 7 (23.3%)	0.500 ^2^
Side (Right vs. Left, *n*, %)	18 (60.0%) vs. 12 (40.0%)	15 (50.0%) vs. 15 (50.0%)	0.302 ^2^
Weight (kg ± SD)	77.4 ± 14.0	82.6 ± 15.1	0.174 ^3^
Height (cm ± SD)	172.3 ± 7.9	173.8 ± 6.8	0.438 ^3^
BMI (kg/m^2^ ± SD)	26.1 ± 4.8	27.2 ± 4.4	0.348 ^3^
Mechanism of injury (*n*, %)			0.329 ^2^
*Sport injury*	20 (66.7%)	25 (83.3%)	
*Traffic Accident*	4 (13.3%)	2 (6.7%)	
*Simple Fall*	6 (20.0%)	3 (10.0%)	
Tobacco Use (Yes vs. No, *n*, %)	10 (33.3%) vs. 20 (66.7%)	12 (40.0%) vs. 18 (60.0%)	0.395 ^2^
ASA Score (*n*, %)			0.298 ^2^
ASA I	20 (66.7%)	17 (56.7%)	
ASA II	10 (33.3%)	13 (43.3%)	

Abbreviations: BMI: Body Mass Index, ASA: American Society of Anesthesiologists, SD: Standard Deviation. ^1^ Mann–Whitney U test, ^2^ Chi-Square Test, ^3^ Student *t*-test.

**Table 3:** Comparison of perioperative characteristics of the patients.

Variables		Tenodesis Group (*n* = 30)	Non-Tenodesis Group (*n* = 30)	*p*-Value
Operation Time (min ± SD)		106 ± 27.4	98.1 ± 22.4	*0.355* ^1^
LOS (days ± SD)		1.5 ± 0.7	1.3 ± 0.4	0.226 ^1^
Additional interventions (*n*, %)				
*MM*	*Medial Meniscus intact*	15 (50.0%)	15 (50.0%)	0.301 ^2^
	*Medial Meniscal Repair*	7 (23.3%)	3 (10.0%)	
	*Medial Meniscectomy*	8 (26.7%)	12 (40.0%)	
*LM*	*Lateral Meniscus intact*	22 (73.3%)	17 (56.7%)	0.187 ^2^
	*Lateral Meniscal Repair*	4 (13.3%)	10 (33.3%)	
	*Lateral Meniscectomy*	4 (13.3%)	3 (10.0%)	
PLT Harvesting Technique (*n*, %)				0.130 ^2^
*Single inframalleolar*		16 (53.3%)	20 (66.7%)	
*Single supramalleolar*		6 (20.0%)	1 (3.3%)	
*Double incision*		8 (26.7%)	9 (30.0%)	
Graft Preparation Technique (*n*, %)				<0.001 ^2^
*Double-stranded*		29 (96.7%)	13 (43.3%)	
*Triple-stranded*		1 (3.3%)	17 (56.7%)	
Graft Diameter (cm ± SD)		8.1 ± 0.5	8.7 ± 0.6	<0.001 ^1^

Abbreviations: MM: Medial Meniscus, LM: Lateral Meniscus, LOS: Length of Stay, SD: Standard Deviation. ^1^ Mann–Whitney U Test, ^2^ Chi-Square Test.

**Table 4:** Comparison of functional knee outcomes between the groups.

Variables	Tenodesis Group (*n* = 30)	Non-Tenodesis Group (*n* = 30)	*p*-Value
Follow-up (months ± SD)	18.5 ± 4.4	16.8 ± 3.4	0.113 ^1^
Preop LKS (score ± SD)	33.5 ± 18.7	59.7 ± 17.2	<0.001 ^2^
Postop LKS (score ± SD)	89.7 ± 13.7	86.7 ± 13.1	0.081 ^2^
Δ Mean (95% CI)	56.2 ± 20.0	27.0 ± 17.8	0.001 ^2^
*p*-value (within group)	<0.001 ^3^	<0.001 ^3^	
Knee Extension Loss (*n*, %)	1 (3.3%)	1 (3.3%)	0.754 ^4^
Knee Flexion Loss (*n*, %)	2 (6.7%)	3 (10.0%)	0.500 ^4^
Knee Flexion Deficit (° ± SD)	3.0 ± 11.4	0.8 ± 2.6	0.711 ^2^
Knee Extension Deficit (° ± SD)	0.6 ± 3.6	0.3 ± 1.8	0.981 ^2^
Lachman Test (*n*, %)			0.949 ^4^
*Grade 0*	20 (66.7%)	19 (63.3%)	
*Grade 1*	8 (26.7%)	8 (26.7%)	
*Grade 2*	1 (3.3%)	1 (3.3%)	
*Grade 3*	2 (6.7%)	1 (3.3%)	
Δ Thigh circumference (cm ± SD)	0.1 ± 0.9	−0.9 ± 3.2	0.679 ^2^
Δ Calf circumference (cm ± SD)	0.5 ± 0.8	−0.2 ± 2.8	0.940 ^2^
Single Leg Hop test (LSI ± SD) *	88.0 ± 12.0	86.1 ± 20.5	0.810 ^2^
Re-rupture (*n*, %)	2 (6.7%)	1 (3.3%)	0.500 ^4^

Abbreviations: LKS: Lysholm Knee Score, LSI: Limb Symmetry Index, SD: Standard Deviation, Δ: Difference, ^1^ Student *t* Test, ^2^ Mann–Whitney U Test, ^3^ Wilcoxon Signed Rank Test, ^4^ Chi-Square Test. Flexion/extension loss = contralateral-referenced deficit ≥ 5° by goniometry. * One patient in each group is missing (*n* = 29 vs. *n* = 29).

**Table 5:** Comparison of ankle functions, donor site morbidity, and complications.

Variables	Tenodesis Group (*n* = 30)	Non-Tenodesis Group (*n* = 30)	*p*-Value	Effect Size (Hedges’ g)
Loss of Ankle Plantar Flexion (*n*, %)	0 (0%)	0 (0%)	NA	
Loss of Ankle Dorsiflexion (*n*, %)	0 (0%)	0 (0%)	NA	
Loss of Ankle Inversion (*n*, %)	0 (0%)	0 (0%)	NA	
Loss of Ankle Eversion (*n*, %)	0 (0%)	0 (0%)	NA	
Loss of Ankle Plantar Flexion Strength	0 (0%)	0 (0%)	NA	
Loss of Ankle Dorsiflexion Strength	0 (0%)	0 (0%)	NA	
Loss of Ankle Inversion Strength	0 (0%)	0 (0%)	NA	
Loss of Ankle Eversion Strength	0 (0%)	0 (0%)	NA	
AOFAS Ankle-Hind Foot Score (score ± SD)	97.3 ± 4.9	95.0 ± 5.5	0.078 ^1^	0.436
FADI Score (score ± SD)	96.8 ± 5.2	95.3 ± 5.5	0.091 ^1^	0.277
Sural nerve injury (*n*, %)	6 (20.0%)	5 (16.7%)	0.739 ^2^	
Infection at the harvest site (*n*, %)	0 (0%)	0 (0%)	NA	

Abbreviations: AOFAS: American Orthopedic Foot and Ankle Society, FADI: Foot and Ankle Disability Index, SD: Standard Deviation, ^1^ Mann–Whitney U Test, ^2^ Chi-Square Test, NA: Not Applicable.

**Table 6:** Comparison of Foot Angles Between Groups.

Angle	Side		Tenodesis Group	Non-Tenodesis Group	*p*-Value	Effect Size (Hedges’ g)
Meary’s Angle	Operated Side	° ± SD	7.99 ± 6.76	4.76 ± 6.32	0.061 ^1^	0.487
		Range	(−9.6–21.5)	(−12.6–14.13)		
	Intact Side	° ± SD	7.05 ± 6.89	5.36 ± 6.11	0.320 ^1^	0.258
		Range	(−8.7–22.0)	(−8.2–17.5)		
Calcaneal Pitch Angle	Operated Side	° ± SD	23.19 ± 5.94	21.41 ± 4.64	0.201 ^1^	0.330
		Range	(12.7–38.1)	(9.0–30.4)		
	Intact Side	° ± SD	23.33 ± 5.43	22.00 ± 4.48	0.305 ^1^	0.265
		Range	(12.1–37.2)	(14.2–32.2)		
Meary’s Angle (Comparison between sides)	Within *p*-value *		0.205 ^2^	0.368 ^2^		
Calcaneal Pitch Angle (Comparison between sides)	Within *p*-value *		0.816 ^2^	0.334 ^2^		

* *p*-value is the comparison of the angular measurements of the operated and intact sides within the same group. ^1^ Student *t*-Test, ^2^ Paired Sample *t*-Test.

**Table 7:** Side-to-side differences in radiographic foot alignment (Δ Meary’s angle and Δ calcaneal pitch angle) and within-group comparison against zero in the tenodesis and non-tenodesis groups.

Variables	Data	Tenodesis Group	Non-Tenodesis Group	*p*-Value	Effect Size (Hedges’ g)
Δ Meary’s Angle	Mean ± SD	0.94 ± 3.97	−0.60 ± 3.58	0.120 ^1^	0.402
	Median (IQR)	0.76 (4.89)	−0.82 (4.94)		
Δ Calcaneal Pitch Angle	Mean ± SD	−0.14 ± 3.35	−0.59 ± 3.29	0.603 ^1^	0.134
	Median (IQR)	−0.60 (2.76)	−0.46 (3.91)		
Meary’s Angle Within *p*-value (vs. 0)		0.205 ^2^	0.366 ^2^		
Calcaneal Pitch Angle Within *p*-value (vs. 0)		0.818 ^2^	0.333 ^2^		

^1^ Student *t*-Test, ^2^ One-Sample *t*-Test.

## Data Availability

The datasets are not publicly available. The de-identified data are available upon request from the corresponding author due to privacy, ethical, and legal restrictions that protect patient confidentiality.

## Abbreviations

The following abbreviations are used in this manuscript:

ACL	Anterior cruciate ligament
ACLR	Anterior cruciate ligament reconstruction
AOFAS	American Orthopedic Foot and Ankle Society
ASA	American Society of Anesthesiologists
BMI	Body mass index
CI	Confidence interval
FADI	Foot and Ankle Disability Index
ICC	Intraclass correlation coefficient
IQR	Interquartile range
LKS	Lysholm Knee Score
LM	Lateral meniscus
LOS	Length of stay
LSI	Limb symmetry index
MM	Medial meniscus
PACS	Picture Archiving and Communication System
PB	Peroneus brevis
PL	Peroneus longus
PLT	Peroneus longus tendon
PL-to-PB	Peroneus longus-to-peroneus brevis
PROMs	Patient-reported outcome measures
ROM	Range of motion
SD	Standard deviation
